# Negative consequences of conflict-related sexual violence on survivors: a systematic review of qualitative evidence

**DOI:** 10.1186/s12939-023-02038-7

**Published:** 2023-10-27

**Authors:** Elena Rubini, Martina Valente, Monica Trentin, Giulia Facci, Luca Ragazzoni, Sarah Gino

**Affiliations:** 1 CRIMEDIM - Center for Research and Training in Disaster Medicine, Humanitarian Aid and Global Health, Università del Piemonte Orientale, 28100 Novara, Italy; 2 Department for Sustainable Development and Ecological Transition, Università del Piemonte Orientale, 13100 Vercelli, Italy; 3 Department of Translational Medicine, Università del Piemonte Orientale, 28100 Novara, Italy; 4 Department of Health Sciences, Università del Piemonte Orientale, 28100 Novara, Italy

**Keywords:** Conflict-related sexual violence (CRSV), Gender-based violence (GBV), Sexual violence, Conflict, Survivor, Sexual and Reproductive Health and Rights (SRHR), Consequences

## Abstract

**Background:**

Conflicts exacerbate dynamics of power and inequalities through violence normalization, which acts as a facilitator for conflict-related sexual violence. Literature addressing its negative outcomes on survivors is scant. The aim of this systematic review was to analyze the qualitative evidence reported in scientific literature and focusing on the negative consequences of conflict-related sexual violence on victims’ physical, psychological, and social dimensions of health in a gender-inclusive and disaggregated form.

**Methods:**

A literature search was conducted on January 13, 2023 on Pubmed, Scopus, and PsychArticles. The search strings combined two blocks of terms related to sexual violence and conflict. A time filter was applied, limiting the search to studies published in the last ten years. Information regarding the main characteristics and design of the study, survivors and their experience, and about conflict-related sexual violence was collected. The negative consequences of conflict-related sexual violence on the physical, psychological, and social dimension of victims were extracted according to the Biopsychosocial model of health. The review followed the Joanna Briggs Institute methodology for systematic reviews and relied on the Preferred Reporting Items for Systematic reviews and Meta-Analyses.

**Results:**

After full text review, 23 articles met the inclusion criteria, with 18 of them reporting negative repercussions on physical health, all of them highlighting adverse psychological outcomes, and 21 disclosing unfavorable social consequences. The negative outcomes described in multiple studies were sexual and reproductive health issues, the most mentioned being pregnancy, manifestations of symptoms attributable to post-traumatic stress disorder, and stigma. A number of barriers to access to care were presented as emerging findings.

**Conclusions:**

This review provided an analysis of the negative consequences of conflict-related sexual violence on survivors, thus highlighting the importance of qualitative evidence in understanding these outcomes and addressing barriers to access to care. Conflict-related sexual violence is a sexual and reproductive health issue. Sexuality education is needed at individual, community, and provider level, challenging gender norms and roles and encompassing gender-based violence. Gender-inclusive protocols and services need to be implemented to address the specific needs of all victims. Governments should advocate for SRHRs and translate health policies into services targeting survivors of CRSV.

**Supplementary Information:**

The online version contains supplementary material available at 10.1186/s12939-023-02038-7.

## Introduction

Gender-based violence (GBV) is an umbrella term used to describe harmful acts perpetrated against individuals through coercion and rooted in gender norms and roles [1]. These revolve around the acceptability of specific attitudes based on the sex assigned at birth, or the perceived sex or gender [2]. Gender norms hinder access to resources and enable power imbalances affecting those who are traditionally attributed less value and those who detach from these norms, creating arbitrary social inequalities [1]. While some groups, such as women and girls, are disproportionately affected by GBV [1], men and boys are also victimized [1, 3]. Other intersecting factors (e.g., connected to gender identity and expression, sex characteristics, sexual orientation, disability, HIV positivity, engagement in sex work, ethnicity, socioeconomic status, age, migration status, religion) amplify these disparities and are often employed by those in power to justify discrimination and enable abuse towards disadvantaged groups [4]. GBV is a public and global health issue [3, 5, 6] that can affect individuals in a variety of forms, including sexual violence (SV), which encompasses any act, comment, or behavior of a sexual nature forcibly or coercively directed towards individuals or targeting their sexual organs [4, 7].

Conflicts magnify dynamics of power and inequalities [4] through violence normalization [8], exacerbating pre-existing forms of GBV, such as domestic and intimate partner violence (IPV) [9, 10], and creating an enabling environment for conflict-related sexual violence (CRSV) [4]. Pre-existing discrimination makes specific groups of individuals more targeted by perpetrators and may increase their chances of being socially excluded after victimization [4]. At times, CRSV is the cause of forced migration, a phenomenon that may increase migrants’ susceptibility to SV while on the move (e.g., forced transactional sex for food, shelter, or protection), at crossing of borders (e.g., forced transactional sex as a toll for passage), as well as in resettlement settings or in host countries [11–13].

CRSV has adverse consequences affecting the overall well-being of survivors and their recovery process [7, 14]. Moreover, humanitarian emergencies, including conflict, cause the weakening or the disruption of health systems, also due to shortages of health personnel or medical supplies [14, 15], hampering access to care. Survivors who migrate as a result of conflict encounter additional difficulties in accessing services on the move and in host countries [11], due to a series of vulnerability factors which typically affect migrants (e.g., legal status, poverty conditions, language barriers) [16], and might be able to access GBV-specific care only in the host country, after weeks or months since violence occurred [17].

Understanding the consequences of CRSV on survivors is crucial considering the high number of ongoing conflicts in which SV is used opportunistically and as a weapon of war [18] due to the climate of impunity [4]. However, the difficulty of collecting data in humanitarian contexts, the sensitivity of the topic, and the vulnerability of the affected population caused by the intersection of consequential traumatic events make academic research in this field particularly challenging and peer reviewed scientific literature scarce. Reviews on CRSV have been previously published, however, they are more theoretical in nature [19], analyze quantitative evidence [20], or focus on specific interventions [21–24]. A qualitative synthesis focusing on individual experiences of survivors [25] and practitioners serving this population and addressing the adverse outcomes of CRSV is needed.

The negative outcomes of CRSV can be understood in light of the Biopsychosocial Model (BPS), first conceptualized by George Engel in 1977 [26], and adapted to a variety of situations, including SV victimization [27]. It encompasses three different dimensions related to physical, psychological, and social aspects of health [26]. This model aims to holistically display the health status of individuals, in this way avoiding focusing only on one sphere (e.g., physical, psychological) and encompassing the influence that the social outcomes of one health condition might carry on the overall well-being and recovery process.

The aim of this systematic review is to retrieve and analyze the published scientific qualitative evidence published in the last ten years, describing the adverse outcomes experienced by survivors of CRSV and affecting their overall well-being and recovery process. Following the BPS Model, the research was guided by the question: “What are the negative physical, psychological, and social consequences of CRSV on survivors?”. Giving an answer to this question will contribute to understanding the extent of the adverse outcomes experienced by victims of CRSV and may inform future research aiming at defining strategies for better response, improving chances of recovery.

## Methods

A systematic review of qualitative evidence focusing on CRSV and exploring its physical, psychological, and social consequences on survivors was performed.

### Data sources and search strategy

This review was guided by the Joanna Briggs Institute methodology for systematic reviews [28]. The Preferred Reporting Items for Systematic reviews and Meta-Analyses (PRISMA) checklist [29] was used to report each stage of the review and findings. A comprehensive literature search was conducted on January 13, 2023, on three databases (PubMed, Scopus, PsychArticles) to identify all relevant peer reviewed studies addressing the consequences of CRSV on survivors. The databases were chosen because they explore health from different perspectives. The search strings combined two blocks of terms, related to SV and conflict (Additional file 1). A broad definition of CRSV was chosen, which highlights the causation occurring between conflict and SV, including that provoking and taking place during the different phases of migration. The research team was composed of researchers with different areas of expertise, namely global health (ER, MV, MT, GF), forensics (SG), and humanitarian aid (LR). After removal of duplicates, the titles and the abstracts of the studies were manually screened and those found not eligible were excluded (ER, MV). The full text of the remaining articles was screened to determine those to be included (ER, MV). The references of the selected articles were also screened to find any other study eligible for inclusion (ER, MV). If discrepancies in the study selection emerged, they were solved after discussion with the whole team.

### Operational definitions

This review of peer reviewed scientific literature relied on the following operational definitions:

- Conflict-related sexual violence: a specific type of GBV that includes rape, sexual slavery, forced prostitution, forced pregnancy, forced abortion, enforced sterilization, forced marriage, forced nudity, forced witnessing, exposure to acts, and any other form of SV that is directly or indirectly (temporally, geographically, or causally) linked to a conflict [4, 18].
- Conflict: this term will be used to encompass not only the International Humanitarian Law definitions of international and non-international armed conflict [30], but also persecution [31], genocide [32], and occupation [33].
- Survivors: people who were subjected to CRSV. They are “frequently an actual or perceived member of a persecuted political, ethnic, or religious minority, or targeted on the basis of actual or perceived sexual orientation or gender identity” [18]. In this study the terms “survivor” and “victim” will be used interchangeably to refer to people subjected to SV; the first term serves to highlight the process of recovery, while the second calls attention to the criminal nature and the severity of acts of GBV. In this review “migrants” will be used as an umbrella term [34] to refer to survivors of CRSV who migrated as a result of conflict.

### Inclusion/exclusion criteria

Studies were included when they met the following inclusion criteria: (a) they were original studies reporting qualitative findings on CRSV (as per the operational definition), (b) they were published in the last ten years; (c) they explored the negative physical, psychological, and social consequences of CRSV on survivors, either from their perspective or that of professionals working with them; (d) survivors were 18 years of age or older when the study was conducted.

A time filter was applied, in order to focus on recently published scientific literature, considering that the conceptualization of CRSV is evolving from a weapon of war [35] to cover also opportunistic and non-combatant perpetrated SV [19, 36–38]. No exclusion criterion was applied to studies published in languages other than English.

Studies were excluded when they did not match inclusion criteria or when (a) they dealt with SV within the military workforce, because we wanted our review to focus on CRSV affecting civilians; (b) they focused on law-related issues (e.g., impunity, transitional justice); (c) the perpetrator was a member of the family or an intimate partner.

Although domestic and IPV are among the most occurring kinds of GBV in peace [7] and during conflict [39], they are not provoked, but rather exacerbated by it. Moreover, IPV and domestic violence are generally grounded in dysfunctional relational interactions where not only cultural, economic, and social aspects have to be considered, but also personal and familial ones [40], which go beyond the scope of the present study.

### Data extraction and analysis

A Google sheet was developed to extract relevant information (Additional file 2). Data included general information about the article, study design, information about the survivors and their experience of CRSV. Data focusing on the consequences of CRSV was extracted (ER, MV) and thematically analyzed following the BPS model, together with other emerging findings.

## Results

**Fig. 1:**
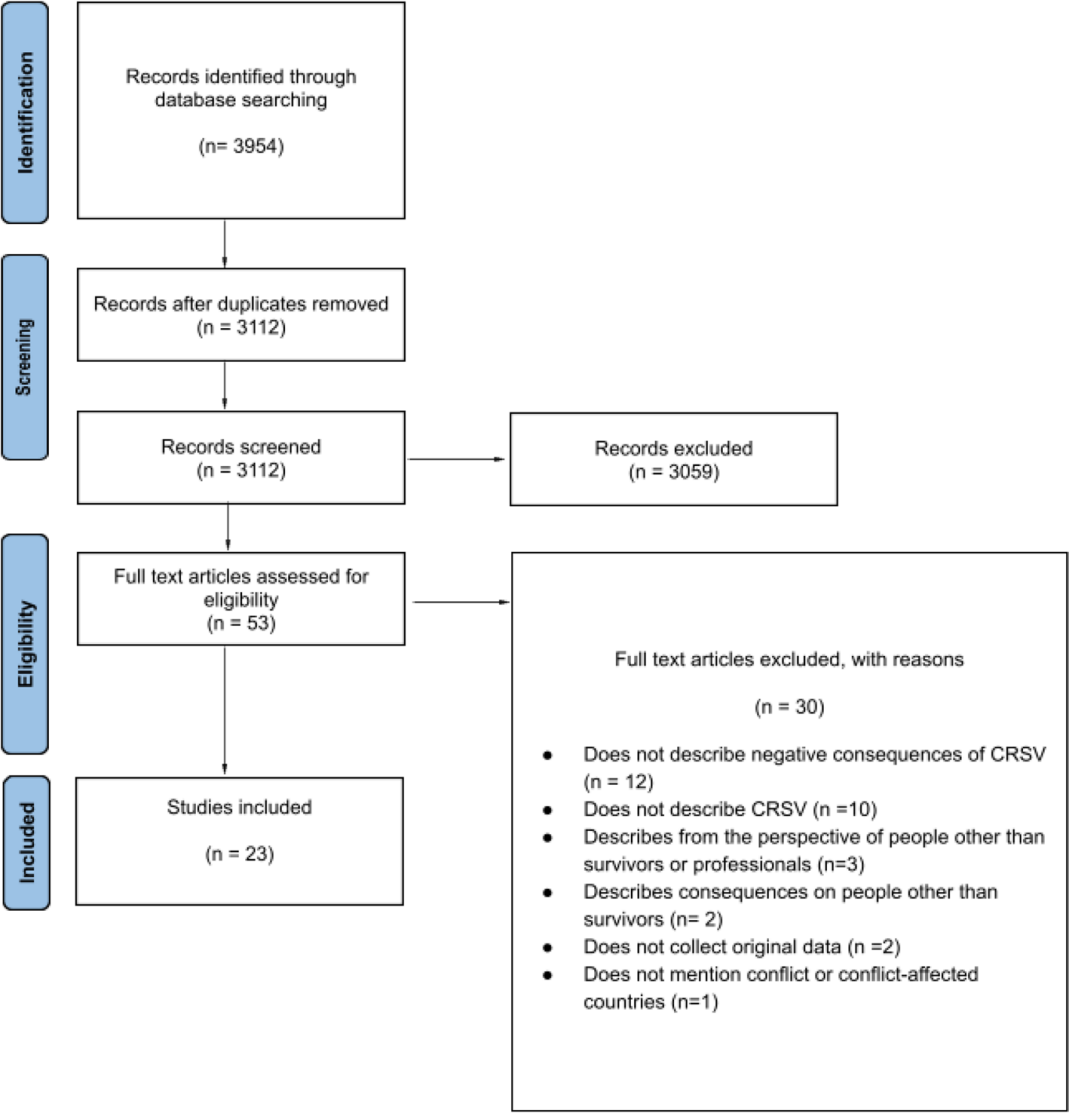
Screening of sources and study selection process

The search returned 3954 results. After removal of duplicates and title and abstract screening, 53 articles were eligible for full-text review. Of these, 23 articles met inclusion criteria. In all the included studies, only qualitative data was extracted. When the study population also included minors, data was extracted only when it was clear that it was not referred to them [41–48]. Since none of the studies reporting the experiences of transgender victims provided details about their gender [42, 49], information about this population will be presented separately from cisgender women and men, together with gender diverse survivors. Detailed information regarding the screening of sources and selection of evidence can be found in the PRISMA diagram (Fig. 1), while a comprehensive overview of the main characteristics of the studies is presented in Table 1 and Table 2 (Additional file 3).

**Table 1:** Characteristics of the included studies

First author, country where the study was conducted, year	Type and location of conflict, and study period as reported by the authors of the studies	Population as reported by the authors of the studies	Consequences of CSRV			Barriers to accessing care
			**B**	**P**	**S**	
Scott, Democratic Republic of Congo (DRC), (2018) [50].	Armed conflict, DRC. October–November 2012.	55 adult women who self-reported sexual violence (SV) and a resultant sexual violence related pregnancy (SVRP).	X	X	X	X
Onyango, DRC, (2016) [51].	Armed conflict, DRC. October–November 2012.	55 adult women who self-reported sexual violence and a resultant SVRP.	X	X	X	X
Kansiime, Uganda, (2018) [52].	Armed conflict, DRC. August 2015- May 2016.	10 Congolese male refugee survivors of CRSV and 6 Ugandan service providers.	X	X	X	X
Palattiyil and Sidhva, United Kingdom, (2015) [53].	Armed conflict. Study period not mentioned.	19 HIV-positive Black African men and women asylum seekers.	X	X	X	X
Yagi, Congo, (2022) [54].	Armed conflict, Congo. od not mentioned.	14 males who experienced SV in the Congolese war.	X	X	X	X
Kohli, DRC, (2013) [55].	Armed and civil conflict, Congo. June-July 2010.	13 adult women survivors of SV who were rejected by any family member; their husbands, community members, and community workers.	X	X	X	
Tenaw, Ethiopia, (2022) [41].	Armed conflict, Ethiopia. May—June 2022.	23 women survivors of rape.	X	X	X	X
Green, Bangladesh, (2022) [49].	Genocide, Myanmar. November 2019- August 2020.	Health care workers who provided care to Rohingya refugees in Bangladesh.	X	X	X	X
Al Issa and Beck, Israel, (2021) [56].	Occupation, Israel. Study period not mentioned.	20 Palestinian women who visited family members in prison.		X	X	
Burkhardt, DRC, (2016) [57].	Armed conflict, DRC. October–November 2012.	55 women who experienced SVRP.	X	X	X	X
Byrskog, Sweden, (2014) [58].	Collapse of State and civil war, Somalia. December 2011—December 2012.	17 Somali born refugee women of fertile age living in Sweden.	X	X	X	X
Keygnaert, Morocco, (2014) [45].	Armed conflict, anti-government protests, crisis, civil conflict. DRC, Cameroon, Congo, Ivory Coast, Mali. Summer of 2008.	154 Subsaharian male and female migrants living in Morocco.	X	X		X
Sager, Sweden, (2016) [59].	Armed conflict, Kosovo. Study period not mentioned.	One heterosexual couple and one woman from Kosovo seeking asylum in Sweden.	X	X	X	X
Weishut, Israel, (2015) [47].	Armed conflict, Israel. Study period not mentioned.	60 testimonies in the Public Committee Against Torture in Israel (PCATI) archive.		X	X	
Woldetsadik, Uganda, (2022) [60].	Terrorism, Uganda. October 2016—March 2017.	30 adult women survivors who lived in northern Uganda.	X	X	X	X
Chynoweth, Bangladesh, Italy, Kenya, (2020) [42].	Persecution, Myanmar. Armed conflict and terrorism, Sub Saharan Africa. July 2018—May 2019.	Rohingya refugees from Myanmar living in Bangladesh, refugees and migrants who had traveled the central Mediterranean route living in Italy, and refugees from DRC, Somalia, and South Sudan residing in urban settings in Kenya.		X	X	X
Dossa, DRC, (2014) [43].	Armed conflict, DRC. July—August 2012.	12 women survivors of conflict-related rape who became pregnant after the assault and gave birth to an infant.	X	X	X	
Haar, Bangladesh, (2019) [44].	Persecution, Myanmar. December 2017, February 2018, March 2018, and July 2018.	114 Rohingya survivors residing in refugee camps in Bangladesh.	X	X		X
Wirtz, Colombia, (2014) [61].	Internal armed conflict, Colombia. June 2012.	35 female internally displaced persons survivors of GBV and 31 service providers.	X	X	X	X
Corboz, Afghanistan, (2022) [62].	Armed conflict, Afghanistan. April-December 2020.	27 adult male survivors of SV, 44 healthcare providers, and 26 community health workers.		X	X	X
Atim, Uganda, (2018) [63].	Armed conflict, Uganda. February 2013—December 2015.	57 female survivors of CRSV perpetrated by parties to the armed conflict.	X	X	X	X
Krause, Uganda, (2015) [46].	Armed conflict, DRC. Study period not mentioned.	28 employees working in the settlement. 35 refugees.		X	X	X
Wirtz, Ethiopia, (2013) [48].	Armed conflict, DRC, Burundi, Sudan, Eritrea, Somalia. January–February 2011.	37 female refugees survivors of GBV, originating from six conflict countries, who received GBV services. 77 FGD participants included health, protection and community service staff providing services to refugees.	X	X	X	X

### Characteristics of the studies

Data about the characteristics of the included studies was extracted and can be found in Table 1 (first author, country where the study was conducted, year of publication, type and location of conflict, study period, population as reported by the authors of the studies, consequences of CRSV, and barriers to accessing care), and Table 2 (study type, methodology, population type, and gender, migrant status and country of origin of survivors).

Eight themes can be recognized among the objectives of the retrieved studies: a) sexual violence-related pregnancy (SVRP), termination and living with children born after SVRP [43, 50, 51, 57, 63]; b) survivors’ help seeking and reporting behaviors, and barriers and facilitators in accessing services [42, 48, 52, 62]; c) CRSV during conflict, flight, migration, displacement, encampment, and in prison [45–47, 56, 58, 59, 61]; d) consequences on physical and mental health and social dimensions [41–44, 47, 49, 55, 60]; e) men survivors and masculinity [42, 47, 54, 62]; f) interventions, tools, and recommendations [43, 45, 48, 55, 62]; g) HIV + (Human Immunodeficiency Virus) survivors [53]; h) general descriptions of GBV [48].

### Characteristics of CRSV

The type of CRSV mentioned in more studies (*n* = 21) was rape [41–51, 53–55, 57–63], sometimes referred to as “sexual assault” [44, 46, 63]. At times, this type of violence was only implicit (e.g., when survivors became pregnant as a result of CRSV) [50, 51, 57]. A summary of the findings connected to the type of CRSV can be found in Table 3 (Additional file 4).

In nine of the studies the location where CRSV occurred was not specified [42, 43, 50, 52, 54, 55, 57, 59, 62]. When reported, attacks took place outdoors [46, 53, 58, 61], in enclosed spaces [41, 44, 49, 61], and in formal or informal places of confinement [47, 51, 56, 60, 61, 63].

For migrants, CRSV occurred at different times during their migration journey in the country of origin [48], with episodes of revictimization in transit [48], and in their host countries [45, 48]. More specifically, sexual assault occurred in conflict [61], at crossing of borders [45, 46, 48], during migration journey [45, 46, 61], in military camps [48], as well as in refugee camps or their whereabouts [46, 61].

Perpetrators of CRSV, when reported, were armed combatants [41, 43–51, 55, 57, 58, 60, 61, 63], supporters of the ruling party [53], rebels [43, 46, 54, 55, 58, 61, 63], prison staff [47, 56], police officers [45–47], secret service officers [47], civilians [44, 46, 48, 58, 61], non-governmental organizations (NGOs) and humanitarian camp staff [46], guides [45], chairmen of gangs [45], and religious and authority figures [48]. Some of the perpetrators were women in a position of power (e.g., police, prison staff, or armed forces) [46, 47, 56] victimizing both women and men. At times survivors’ acquaintances enabled CRSV [48].

### Conflict-related non-sexual violence

CRSV takes place in a context characterized by widespread violence. Apart from CRSV, mentioned personal offenses included physical violence and threats [41, 43–46, 48, 49, 53, 54, 56, 58, 60, 61, 63], war-related violence [58], violence specifically targeting migrants [44, 59], persecution [44, 47], discrimination [44, 45, 47, 56], as well as forms of GBV separated from our operational definition of CRSV [41, 45, 46, 48, 50, 51, 53, 55, 56, 58, 61, 63]. Two liminal situations appeared, in which it is not possible to understand the degree of agency exercised by the women involved, that is when consent for marriage was given only to acquire protection [48], and when sex work became the only possible mean to financially sustain themselves [61]. Property offenses were also common [44–46, 49, 55, 61, 63]. Three studies explicitly mentioned violence as a reason for fleeing [46, 53, 58].

### Negative consequences of CRSV

The negative outcomes experienced by participants in the included studies as a result of CRSV will be reported as divided into three components (physical, psychological, and social) according to the BPS model [26]. The findings will also be gender disaggregated, explaining those affecting cisgender female survivors and cisgender male survivors. Barriers to access to care were an important emerging finding and were conceptualized and understood in this paper as per the access to care framework advanced by Levesque et al. (2013) [64]. They will be presented according to the BPS model (e.g., elements that negatively affect access to physical and psychological care, and social aspects that hinder access to care) and in a gender disaggregated form. With regard to transgender and gender diverse populations, findings in the included studies were scant and therefore only barriers to access to care were explored. A summary of the type of consequences experienced by survivors, as well as of the barriers to accessing care they faced, can be found in Table 1.

### Physical consequences of CRSV

#### Cisgender women

Many of the findings were connected to the Sexual and Reproductive Health (SRH) of survivors.

Regarding their reproductive health, survivors experienced pregnancy, sterility, or loss of infants during gestation. In 13 studies pregnancy was mentioned as an outcome of rape [41, 43, 45, 48–51, 55, 57, 58, 60, 61, 63]. One study about Rohingyas mentioned the phenomenon of excess births because of SV [49]. In two studies, sterility provoked by SV was mentioned as affecting survivors [55, 59]. One of the studies described a pregnant woman being raped by rebels, who on the same occasion forced an abortion on her, provoking loss of infant [61]. SVRP caused women to suffer further GBV when their husbands forced them to have an abortion [50, 51] or attempted to force them to do so [55]. When women were held captive in a situation of sexual slavery, they were ridiculed and beaten by armed combatant perpetrators when they disclosed their pregnancy status [51].

Genital disturbances were another finding frequently mentioned in the studies. These included injuries on genital and pelvic area [41, 59, 61], fistulae [55], vaginal and anal tearing [45], lacerations [44], vaginal bleeding [41, 48, 53], vaginal infections [60], vaginal discharge [41, 49], and abdominal pain [45, 53, 60]. In other circumstances providers noticed laceration scars on the perineum during medical examination [44, 49].

Sexually transmitted infections (STIs) were also mentioned [41, 45, 48, 55], in particular HIV [41, 45, 48, 53, 55, 63].

Urinary issues, specifically urinary tract infections [41, 60], micturition issues [41], and bladder dysfunction [60] were also reported.

Blunt force trauma inflicted on other erogenous sites, such as breasts, caused survivors to develop infections [41]. Some participants experienced breast mutilation [44]. This quotation clearly shows the extent and the variety of injuries experienced by one survivor:

“They raped me not only on the genital but also rectal. I lost consciousness. My breast was beaten and infected as a result. I was also exposed to several infections of the urinary and reproductive organs. I can’t sit to pee because my genital and pelvic areas were injured. As a result of the assault, I was diagnosed with HIV positive.” (Survivor) [41]

The experience of CRSV affected the general health of women, causing in the short-term difficulty to walk [45], loss of consciousness [41, 45, 49, 53], external hemorrhage [45], septicemia [49], and coma [53]. In the long term, it provoked general pain [55], musculoskeletal pain [41], and backache [63] because of the injuries sustained, as well as psychosomatic symptoms due to psychological trauma such as chronic chest pain [60], heart palpitations [43], sickness [45, 55], weakness [43], stomachache [49], and headache [55]. Some participants also disclosed fatal outcomes of other women subjected to SV due to the injuries [44], suicide [43], or murder [44–46, 49], at times associated with mutilations [44].

Acts of non-sexual violence perpetrated during sexual assault caused unspecified injuries [45, 49, 55], head injuries [48], blunt force [44], or sharp edge [44] trauma to unspecified regions of their body. In some studies participants reported being shot before violence occurred, and one of them risked her leg being amputated due to firearm wounds [53]. One survivor reported being beaten by her aggressor, which caused bone fractures [44]. In other cases, women were stabbed after rape occurred [44], leaving them with penetrative stab injuries. Participants reported being left to die or unconscious by perpetrators [49, 53]. The physical impairment [45] derived from CRSV or co-occurring violence reduced some of the women’s work performance [41].

#### Cisgender men

Cisgender male survivors experienced urinary system and bowel issues, in particular loss of control of urinary and anal sphincter muscles [52, 54]. Other participants mentioned suffering from hematuria [54] or hemorrhoids [54].

As to genito-anal disturbances, some of the survivors sustained blunt [47, 54], electric [42] force, or mutilating injuries [54], as well as wounds due to the use of irritative agents [54]. The consequences of sexual assault hindered or disenabled their possibility to have an active sexual life with their partners [54].

The overall wounds sustained, and loss of strength provoked by the attack prevented some of the survivors from working for a period of time after violence occurred [52, 54]. Survivors were left with permanent wounds, scars, and bruises [54]. Violence was sometimes perpetrated in order not to leave physical signs [47]. Cisgender men subjected to CRSV suffered from general pain, back pain, weakness, loss of balance, bloody noses, loss of appetite, and stomachache [54].

### Psychological consequences of CRSV

#### Cisgender women

Participants shared numerous psychological consequences of CRSV, and the sum of these symptoms has been generally defined in some of the studies as trauma [46, 49, 59] or post-traumatic stress disorder (PTSD) [46, 49, 53]. Survivors reported feeling distressed [44], terrified [44, 53] and fearful [41, 44, 45, 55], for their future [41], of gossiping in the community [58], and for their lower chances to become wives [48, 55]. They had fear that the assault might happen again [41], of people wearing the same clothes as perpetrators (e.g., ranger clothes) [41], of the location where the assault occurred [41], of the dark [53], of groups of people [53], that the perpetrator might come back to re-victimize them [41, 48], which prevented them from reporting SV [46], or were concerned of other people targeting them for further harm [48]. Migrant participants reported being afraid of being attacked while fleeing from their country [46].

Participants also shared fear of negative reactions from family and community [48], including rejection [61], and of the stigma associated with people living or presumed to be living with HIV [48]. Single women were concerned about being victim-blamed and labeled as promiscuous [48]. Exclusion from the community provoked by stigma caused psychological suffering [63] and self-stigmatization [55], leading one survivor to report:

“The woman who has known another man has no more value” (Survivor) [55].

Sometimes victims were emotionally abused by their husbands due to their experience of CRSV [60]. Moreover, trauma provoked by CRSV caused loneliness and hindered their relationship with other people [44]. Migrants were concerned of possible negative repercussions in the host country if SV became public [48].

Survivors felt detached from their previous self [41, 43, 55], thought that their lives had been destroyed by SV [43], felt ashamed [41, 45, 48, 55, 58, 61], worried [53], isolated [55], humiliated [53, 56], and reported low self-esteem [41]. They were sad [55], also because they felt or had the desire to keep SV a secret [43, 58], were constantly crying [49, 55, 56], or not showing any emotion [49], overthinking [55], and reliving the experience of SV [41, 43, 53, 55, 60], also through flashbacks [46, 53] and intrusive thoughts [44]. They experienced persistent arousal [45, 49, 53] and hastiness [46, 49], had sleeping difficulties [41, 44, 46, 53, 56], nightmares [43, 46, 60], or were in a lethargic state [41, 53], could not talk [49, 53, 56] or move for a period of time after the assault [56], or felt exhausted [56]. Survivors perceived their daily lives as oppressive [43] and lacked motivation in everyday activities [43]. They had trouble focusing [41, 53], and were forgetful [41], which hindered their working capacities [41], as well as their ability to care for their children [60]. Conversely, some reported feeling nervous [56], or angry [56]. Due to security concerns, they repeatedly checked doors [41] and experienced anxiety [41, 44, 49, 53, 56, 60]. Some of them used alcohol consumption to try and relieve symptoms [46, 53], developed eating disorders [41], depression [41, 44, 49, 56, 60], and had suicidal [41, 43, 53, 60], or self-harm thoughts [60], or even attempted to commit suicide [43].

All these symptoms, together with amnesia [53], prevented cisgender migrant women survivors from giving a coherent and detailed account to border authorities, and from showing any emotion while recalling those events, which were dismissed as fabrications causing the rejection of their asylum applications [53]. One study reported that participants started shaking and crying and felt nauseous and dizzy at recalling the memories of the violence with researchers [53]. Migrant women survivors feared deportation, which also caused reliving of traumatic memories such as being rejected from their family after CSRV occurred [53].

SVRP was associated with numerous negative outcomes regarding the mental health of survivors. Fear of social rejection and of stigmatization or of rejection of children born after SVRP were a driver for women who decided to terminate in order to preserve marital and family relations [50]. Some of the victims felt upset, hopeless, weak, restless, nervous, and suicidal when they discovered their pregnant status [43, 50, 51], fearing God’s punishment [50] or legal repercussions [51] if they chose to terminate. Some survivors decided to continue the pregnancy to term precisely because they didn’t want to go against their religion (e.g., Catholic) or God’s will, or since they thought it could carry a positive meaning to their lives [50] or owing to the fact that they perceived abortion as a sin [57]. In one study, SVRP was given a different status compared to a “normal” pregnancy and its termination was not seen as badly [50]. Disclosure to confidants influenced women’s decision-making [51]. Generally, survivors lacked trust or trusted only a limited group of people [51].

Some participants expressed concern surrounding having a child whose father was an armed combatant or of different ethnicity, worrying about her or his future integration in the community or possible discrimination [50, 58, 60, 63]. Some of them feared for their children's safety if their experience of CRSV became public [48]. Other women, who were already mothers, were worried about the possibility of dying during termination, their children becoming orphans [57]. Some of the survivors had positive or mixed feelings surrounding the baby [43, 50], while others considered the fetus as “a curse” or “the devil” and expressed relief after terminating the pregnancy [50]. Conversely, some of the victims expressed regret after abortion [50]. The preservation of a woman’s honor following SVRP also had an ambivalent connotation: terminating gave to some the possibility to focus on their needs, while others saw abortion as a killing and a loss of dignity [50]. Some women rejected the pregnancy and perceived the baby as an additional punishment [43]. Others lacked the desire to have other children in addition to those born after CRSV [43].

#### Cisgender men

The included studies mentioned shame [42, 45, 47, 52, 54], stress [44], humiliation [47], dishonor [47, 62], loss of dignity and reputation [62], self-stigmatization [42, 62], self-victim blaming attitudes [42, 62], and terror [44]. Fear [44, 45, 52], including of exclusion and stigmatization [42, 46, 62], of re-victimization [54], of walking the same path they did when they were assaulted [54], of emotional and physical abuse [62], of extortion [62], of being killed or of receiving other threats from their family members or the community [62], and of additional violence from the perpetrator [42, 46, 62] were also mentioned. Migrant survivors reported fear of being attacked while they were fleeing [46].

Other psychological symptoms of trauma [46, 49, 52, 54] were hastiness [46, 49] and restlessness [45], inability to sleep [44, 46], nightmares [46], as well as flashbacks [46] and intrusive thoughts [44]. Some of the survivors tried to relieve them by increasing their alcohol consumption [46]. Survivors suffered from depression [44, 49], with some of them continuously crying [49], and anxiety [44, 49]. The traumatic experience of CRSV caused relational difficulties with other people [44], shyness [52], and loneliness [44, 52] and hindered their ability to speak [45, 49] and to show emotions [49]. Two of the studies explicitly mentioned PTSD [46, 49, 54].

Survivors suffered because they perceived a clash between gender expectations and their experience of SV [42, 54], which made them feel damaged, destroyed and broken, and reduced to a “de facto female” [54]. Some of them also feared being labeled as “homosexuals” [47, 52] and being subjected to homophobic acts and comments [52]. In one study, some gay refugee survivors reported perceiving SV as a “deserved punishment”, self-blaming themselves [42], and being also concerned about the exposure of their sexual orientation, which could compromise their security and that of their families [42]. Heterosexual survivors instead feared being perceived as “gay” or were afraid that rape might have “turned them gay” [42].

Participants reported living in a state of loss and grief [54], feeling stuck and with a sense of hopelessness [54] and failure [54], also connected to their felt “lack of sexual force” [54]. They felt sad [54] and powerless [54], these emotions carrying a negative influence on their marriage life [54].

### Social consequences of CRSV

#### Cisgender women

For many survivors the decision to continue or terminate a pregnancy relied consistently on the perceived and anticipated attitudes from their spouses, members of their family and communities [49, 50] as well as on the advice given by trusted confidants, usually a female peer [51]. Mother-in-laws frequently asked the women to have an abortion [51]. Some of the survivors were forced by their husbands to terminate [50], and those who decided to continue the pregnancy were rejected [41, 50, 57]. To avoid this, some victims pretended the father of their baby was their husband [58]. Due to the financial difficulties experienced by women after rejection, some had to resort to prostitution [51]. Abortion-related stigma affected those who terminated [58]. In some contexts, termination was rare because it was perceived in a negative light [58]. In some instances, family members other than the husband supported the decision to continue the pregnancy [50] or advised the women not to terminate following cultural and religious beliefs [49, 51]. Returning from abduction with a child was at times used by their partners as a justification of emotional, physical, verbal, and sexual abuse, as well as of rejection [63].

In most cases, communities lacked compassion and did not provide emotional support to women subjected to CRSV [43]. Conversely, survivors suffered stigmatization [43, 46, 48, 55, 58, 60, 61, 63], humiliation [43], victim blaming attitudes [41, 43, 48, 55], discrimination [55], mocking [55], sometimes because they became beneficiaries of donors or of the government [41], exclusion [41], insults [60], and isolation [48, 55]. A survivor described her experience of stigmatization and victim blaming from family members with these words:

When my older brother learned I had been raped, his first reaction was to throw me out of the house because I had shamed them. My brother said the family had done everything to raise me properly, and now I had paid them back by letting myself be raped. For the family, it was as if what had happened was my fault [43].

Risk of stigmatization and isolation prevented many women from disclosing [58]. In some cases, mothers of survivors asked them not to disclose to their husbands experiences of CRSV [56], because this could lead to fights and divorce [56, 57]. Sometimes the inability to move on from the traumatic experience detached survivors from their family and community [60].

Survivors internalized community perceptions of CRSV as a transgression from social norms [63] provoking the loss of a woman’s worth [43, 55, 58] and detachment from traditional femininity ideals [43]. Unmarried women subjected to SV were perceived as “used” and had difficulties finding a husband [58, 63]. In some instances, survivors were rumored to be friends with [55, 58] or the “wife” of [41, 43] aggressors, or were assumed to be positive to HIV [61], causing social exclusion. Survivors reported that those who were raped more than once [55], by more than one perpetrator [55], that experienced SVRP [43, 55], or whose rape was witnessed by someone [55] had more probability to be rejected. Husband’s rejection was influenced by pressure from members of the family and of the community [55], who sometimes made them believe that the perpetrator would have come back to kill those who supported the woman [55], or that she might have turned into a rebel and a murderer [60, 63]. In some cases, the community perceived the attack as an assault on themselves [41], while families felt that as a dishonor [43].

Disclosing CRSV victimization to the spouse provoked changes in communication [55], in showing affection [55], in family roles [55], and caused misunderstandings [55], negative changes in the relationship between husband and wife [55, 58], or in the family structure [56], and affected the way children of the couple [55] or children born after CRSV [43] were treated by the spouse. Women survivors of CRSV were chased away or abandoned by their husbands [43, 55, 57, 60] and were rejected by their families [43, 55, 60] and communities [43, 60], sometimes together with their children [53, 55]. Some participants reported being insulted [55], or otherwise abused by their husbands [63], that the experience of SV was used against them during arguments [60], or that men rationalized rejection claiming that their wife “got sick” as a consequence of CRSV [55]. Husbands equated SV with adultery and for this reason no longer considered themselves married to survivors [55]. This served as a justification for men to remarry and become polygamous [55].

Regarding the sexual and intimate life of the couple, one study mentioned mutually opposite attitudes towards survivors: husbands either refused sexual intercourse with their wife or that constituted the only interaction they expected from them [55]. Some husbands took back into their home their wives after rejection [43, 55] but made them live in a climate of emotional and physical abuse [43]. Moreover, it has been shown that some children could be ashamed of the fact that their mother was raped, having sometimes witnessed the assault, and for this reason detached from her [55]. In some instances, members of the family or of the community supported survivors [55].

CRSV had lifelong consequences on livelihood and earnings [63]. Women affected by rejection lived without shelter and in poverty [55], also because they had difficulties working [55, 60, 63] or continuing their education [63]. Sometimes this was exacerbated by the fact that family members could take survivors’ possessions or did not allow them to work on family land [55]. Poverty also led to malnutrition issues, in some cases affecting breastfeeding [55]. In some instances, women still living with their husband were refused financial support from him [55, 63].

Survivors reported high prevalence of threats [63] and personal and property offenses from members of the community (e.g., disappearance of family members, housebreaking, land grabbing, livestock theft, physical attack or assault, serious physical harm to a child, general theft, poisoning of a family member, rape, or sexual assault) [63], as well as physical and social attacks from family members [63]. Sometimes abuse was provoked by the fact that women didn’t want to give to male members of the family the reintegration sum received by NGOs [63].

As a defensive strategy, survivors could decide to leave the family home due to emotional abuse [60], to enter a relationship after rejection from their family [63], or to isolate themselves [43]. Others moved away or fled abroad [58, 59]. From a legal perspective, lack of forensic documentation of the physical and psychological consequences of CRSV negatively affected the outcome of survivors’ asylum application process, preventing them from starting a new life in the host country [53].

#### Cisgender men

Survivors experienced stigmatization [42, 46], social exclusion [42, 45], and humiliation [42]. Participants reported discrimination [52] and being forced to stay silent [54]. Moreover, the injuries caused by CRSV created embarrassment in public situations because survivors could no longer control their sphincter muscles [52].

There was a perceived oversight of male victimization [42]. The experience of CRSV clashed with traditional notions of masculinity [42, 47, 52, 54], correlated to men’s capacity to fight off abuse [52]. Male survivors were no longer considered men and respected in their family and community [52, 54], as reported by one survivor:

“I had problems in my family because my wife…when I told her that I was a male survivor, she told me, ‘I don’t deal with homosexuals.’ She left.” [52]

Another notion connected to masculinity is the social norm that identifies men as the head of the household [52]. Due to the injuries, or because of medical treatment, survivors could no longer work [52, 54] and for this reason their family had limited financial resources [52]. Sometimes this situation created a reversal of roles in the family system (e.g., the wife financially sustained the family) [54]. Injuries sustained hindered the possibility for many to have a healthy and regular sexual life [54]. The detachment from stereotypical ideals of male sexuality had a negative impact on recovery and could lead to the disruption of the family [52, 54]. For all these reasons, sexual victimization was reported by some participants as an act of symbolic emasculation [54].

From a legal perspective, lack of forensic documentation regarding the physical and psychological consequences of CRSV hindered migrant survivors’ asylum application process in the host country [53].

### Barriers to accessing care

#### Cisgender women

Regarding their physical health, lack of or low-quality emergency care services [41, 61, 63] as well as low clinician-to-patient ratio [48], sometimes connected to conflict-induced healthcare disruption [58, 59], prevented survivors from accessing care. In other cases, survivors experienced delays [61] or lack of effective treatment [63]. Delays and refusal of care from healthcare workers connected to the persecution of Rohingyas caused worsening of injuries, and sometimes resulted in permanent disability [44]. While in one study providers said that survivors did not follow referrals, and that they sought care only for physical trauma [61], in two studies women affirmed that, apart from the care immediately received after rescue from abduction, they did not have access to any kind of follow-up care [48, 60].

Avoidance of care-seeking behaviors were connected to the inability to afford care [48, 60], in some instances due to minimal or no health and protection services [48], and lack of knowledge on how to access care [60], also due to the secrecy survivors attributed to the experience of CRSV [58, 60, 61]. Unawareness of the health risks connected to untreated injuries [48], or of available services [48], as well as the belief that GBV was a normal part of women’s lives or that no one else had experienced this crime [48] were other reasons why women did not seek care. Some participants reported having traveled long distances to access specialized care due to severe trauma [61], while for others, lack of transportation was a barrier to accessing care [48].

In some contexts, migrants had to rely on NGOs and social support because they were not granted official care [45] or encountered language barriers which hindered their access to services [48]. Lack of documentation of medical forensic evidence of the abuses endured by migrant survivors led to border authorities not believing them and rejecting their case, assuming that their stories were fabrications, despite being shown the scars from the injuries sustained [53].

Access to specific post-sexual assault services was sometimes subjected to the official disclosure [61] or reporting [46] of the violence to authorities. Other problems women experienced were connected to emergency care personnel not asking them questions related to the cause of the injury during medical examination, treating only their manifested symptoms without investigating if they originated from CRSV, due to lack of GBV specific training [48] or of SV specific services [45]. In one study, providers reported that survivors disclosed their experience of CRSV while accessing care for other kinds of health issues [49]. When male survivors tried to access care through women-only post-sexual assault services, this compromised the ability of female survivors to feel safe, causing avoidance of care-seeking behaviors [42].

Some survivors decided to terminate their SVRP [41, 50, 51, 57, 58, 60]. In some instances, medically induced termination was available [41, 57], however, even in this context, some providers refused to perform it, or did not follow evidence-based methods [57]. A number of women had to rely on unsafe, traditional methods (e.g., herbs, quinine, injections), due to restricted access to legal abortion [49–51, 57, 58], and some of them failed to terminate or decided to continue their pregnancy because they were unaware of how to self-perform this procedure [49, 57]. The need for comprehensive post-abortion care was mentioned in one study [49].

As for what concerns the mental health of cisgender women survivors, four studies reported lack of psychological support services [48, 58–60]. Negative consequences on their mental health such as shame, fear, and anxiety, or concerns about confidentiality hindered care-seeking behaviors [41, 48, 58, 60, 61]. In some instances, survivors expressed specific fears, for example that providers would have shown shock or surprise hearing their accounts [48], preventing them from seeking care. Some of the women avoided seeking treatment because they were concerned that children could have been kidnapped if left alone at home while the survivor was seeking treatment [48].

Stigma and victim blaming attitudes from providers constituted social barriers to care [49, 61].

#### Cisgender men

There was limited information about the availability of post-CRSV services and where to find them [42, 52]. Distance to facilities, lack of transportation, and climatic conditions hindered care-seeking [52]. Some survivors expressed difficulties in accessing care after SV and in the long term [54]. As a result, some of them used traditional medicines for finding temporary relief from the symptoms (e.g., tea, hot water) [54]. Financial cost of transportation and of services was also a deterrent for seeking care [42, 54, 62]. As a consequence of the loss of control of urinary and anal sphincter muscles, some of the survivors had to wear adult diapers, which were difficult to afford [52].

Some participants found it hard to categorize their experience as “sexual violence” (e.g., in cases of forced witnessing or genital violence) [42]. In other instances, they were unaware of the entirety of the services or of specific treatment (e.g., post-exposure prophylaxis) or didn’t think that recovering was possible [42]. Narrow definitions of what constituted SV, together with criminalization of same-sex sexual interactions also hindered access to care [42].

Moreover, health providers lacked awareness and training on SV against men and public health facilities did not provide services specific for them [42, 52, 62], or the demand outweighed supply [42]. Protocols for treatment of sexual violence-related issues in male survivors were not implemented [62]. Male survivors have few entry points and sometimes try to access women-oriented services [42]. Cisgender male survivors encountered discomfort in disclosing to female healthcare staff, who in some contexts were the only ones trained to treat survivors of SV [42], sometimes preventing disclosure [52]. High turnover of staff [52], as well as breaks in confidentiality and privacy [52, 62] were also barriers to care-seeking. The quality of care was low [52, 62] or variable [42], and survivors were referred to infrastructures located far away from each other [52], or the referral system was non-existent or poor [42]. Participants reported having to wait long hours for access to care [52]. Lack of food made it hard for them to adhere to medication regimens [52].

For migrant survivors, language barriers created communication issues [42], delayed help-seeking and, with the use of an interpreter, could result in a break in confidentiality [52]. Some migrant survivors were reluctant to seek care for fear of losing their ability to access protection or other services or benefits, or because they were concerned of being arrested or deported while accessing care [42].

As for what concerns the mental health of cisgender male survivors, participants to the studies expressed fear of retaliation from the abusers [42], of providers reporting their case [62], of secondary victimization from healthcare workers [62], of being sexually assaulted by them (Corboz), and of lack of confidentiality [42, 62]. They also reported fear of disbelief, of being labeled as “homosexuals” and of being targeted by homophobic providers [52].

Regarding barriers connected to the social dimension of victims, when survivors decided to seek care, this could create disharmony and eventually lead to break up in the couple [52]. SV is culturally perceived as a taboo or a curse, which prevents reporting abuses to health care providers [52] and causes avoidance of care-seeking behaviors [62]. Some healthcare workers refused to provide care to survivors due to racist, xenophobic, and homophobic attitudes [42]. Survivors with disabilities were overlooked, their experience denied due to stereotypes connected to their condition and sexuality [42]. Participants reported that providers did not believe victims, or they failed to recognize and document some forms of SV (e.g., witnessed, and genital violence) [42]. Survivors reporting sexual assault to providers often encountered victim blaming and homophobic attitudes (e.g., being advised to “stop doing bad things”) [62] or were assumed to be sex workers and stigmatized [62]. Participants who disclosed to researchers their homosexuality reported sustaining verbal and sexual abuses from healthcare providers, especially when their gender expression was non-conforming to stereotypical masculinity standards [62]. Health providers lacked empathy, made humiliating comments towards victims, and ridiculed them [42].

A number of cisgender male survivors encountered heavier barriers. In one study conducted in Afghanistan before the 2021 Talibans’ takeover, some members of the healthcare staff disclosed their support of rape myths (e.g., the impact of SV on men is lower than on women, men rape other men due to “uncontrollable sexual desires”) [62]. Misconceptions of providers, who believed that penile-anal rape might have “turned gay” heterosexual survivors, or that they were no longer men were also reported [42]. Criminalization of same-sex interactions in Bangladesh and Kenya, where “carnal intercourse” can lead to life imprisonment, prevented survivors from seeking care [42]. Forced reporting, without the survivor’s consent, of cases of anal rape (categorized as “sodomy” and a criminal offense in Afghanistan) constituted another barrier for seeking care [62]. Lastly, one survivor reported one of his friends being re-victimized and sexually abused by a provider while receiving post-rape care [62].

#### Transgender and gender diverse population

Regarding their mental health, transgender refugees were concerned about the exposure of their gender identity, which could compromise their security and that of their families [42].

Restrictive policies and narrow definitions of SV, together with providers’ transphobic attitudes, including refusal of care, affected their access to health facilities [42].

## Discussion

This systematic literature review explored the negative physical, psychological, and social consequences of CRSV on survivors, as well as barriers to access to care. Across the 23 included studies, 18 reported negative repercussions on survivors’ physical health [41, 43–45, 48–55, 57–61, 63], all of them highlighted adverse psychological outcomes, while in all but two [44, 45] participants reported unfavorable social consequences. Overall, 19 of the included studies presented barriers to accessing care of survivors of CRSV [41, 42, 44–46, 48–54, 57–63].

The most frequently reported physical consequences for cisgender women were pregnancy [41, 43, 45, 48–51, 55, 57, 58, 60, 61, 63], genital disturbances [41, 44, 45, 48, 49, 53, 55, 59–61], and STI infection [41, 45, 48, 55], including HIV [41, 45, 48, 53, 55, 63]. For cisgender men, less varied but invasive injuries (e.g., mutilating ones or caused by blunt or electric force, or irritative agents) [42, 47, 54], and urinary or bowel issues [52, 54] were mentioned more often.

The most frequently reported psychological outcomes, widely affecting both cisgender women and men, were manifestations attributable to groups of symptoms characterizing PTSD (e.g., intrusive memories, avoidance, negative thoughts, arousal) [41–49, 52–56, 58, 60–62, 65], especially fear and negative feelings towards themselves.

The social consequences highlighted in more studies were for cisgender women stigma [43, 46, 48, 55, 58, 60, 61, 63], victim-blaming attitudes [41, 43, 48, 55], and various forms of rejection and exclusion [41, 43, 48, 50, 53, 55, 57, 60]. Financial difficulties were also evident [51, 55, 60, 63]. For cisgender male survivors, clash with gender expectations [42, 47, 52, 54], stigma, discrimination, and social exclusion [42, 46, 52] were the most disclosed.

Even though these findings have been presented into three separate spheres for clarity purposes, they are deeply interconnected, with multiple feedback loops between one another, and barriers to access to care exacerbate the negative impacts. The interconnections across the three spheres are particularly visible when considering SVRP. Cisgender women survivors reported negative psychological outcomes connected to discovering their pregnancy status [43, 50, 51], as well as to having to decide to continue or to terminate. In both cases, women feared negative social repercussions due to the different ethnicity of the baby, or because the father was an “enemy” armed combatant [48, 50, 58, 60, 63]. Termination was associated with fear [57] fueled by barriers to access to care provoked by restricted access to legal abortion or providers’ beliefs [49–51, 57, 58], and carried a negative reputation in the community [49, 51, 58]. SVRP created a never-ending cycle of stigma, affecting both those who carried it to term and those who terminated [41, 50, 57, 58]. Moreover, pregnancy was a major cause of abuse and rejection in the family [41, 50, 57, 63], which elicited financial difficulties for survivors [51].

The findings of the present study shed light on the importance of avoiding considering GBV and Sexual and Reproductive Health and Rights (SRHRs) as separate domains [66], for various reasons. Firstly, the consequences of CRSV are deeply interconnected with SRH from a physical, psychological, and social perspective. Secondly, CRSV constitutes a violation of human rights, including Sexual and Reproductive Rights (SRRs) (e.g., choose one’s own sexual partner, engage in consensual sexual relations, make free and informed decisions concerning reproduction) [66]. Thirdly, from a social perspective, in the included studies, the spouse, family, and community of survivors, and worryingly some of the healthcare workers, equated CRSV to sexual intercourse [42, 43, 55, 58, 62, 63], and sanctioned it as a transgression from acceptable social norms surrounding sexuality [66].

The gendered nature of this crime was expressed by the different kinds of SV and specific negative outcomes cisgender female and male survivors reported in the included studies, the most striking example being how CRSV affected their sexual life. In fact, women tended to disclose symptoms of SRH issues [41, 44, 45, 48, 49, 53, 55, 59–61] or relational consequences (e.g., having their husband refuse any sexual interaction, or conversely being reduced to a sexual object) [55], while men focused more on their reduced strength for engaging in this activity and the consequent “status loss” and clash with gendered expectations, which could lead to couple separation [54].

Heteronormativity refers to the belief that heterosexuality is the norm, and that men and women have specific, binary, and complementary gender-based roles [67]. The first are assumed to have high sexual interest and to be dominant, while the latter are taught to respond to men’s proposals but to avoid initiating sex [67]. This is reflected in the Traditional Sexual Script [68], which relies on cultural, interpersonal, and intrapsychic scripts that define what is socially acceptable in terms of sexuality [68], and subsequently normalize behaviors where men claim sex from women, which in turn are expected to comply. In respect to the focus of the present study, these gender norms and stereotypes are particularly harmful because they justify sexual coercion and violence in two gender-specific ways. The experience of cisgender men survivors of CRSV detaches from heteronormative sexual norms expecting men to have sexual interactions exclusively with women [69] and to have an active and dominant sexual behavior [70]. Conversely, for the community, victimization of cisgender male survivors mirrors the subordinate condition which is usually attributed to women in heterosexual relationships in terms of power differentials [71]. For gay male survivors, this effect intersects with another level of vulnerability connected to homophobia [42, 62, 69]. However, heterosexual men in the included studies were or feared being subjected to homophobic attitudes and acts from the community or providers, and internalized these beliefs, which hindered recovery [42, 52, 62]. On the other hand, cisgender women survivors infringe heteronormative sexual norms because SV detaches them from notions of purity and monogamy, hindering their “worth” [43, 55, 58], which is decreased by sexual activity and by a perceived lack of defensive stance [72]. Unequal gender roles lead to a normalization of coerced sex [73], and create an environment where the use of emotional, psychological, or other kinds of violence in order to exert sexual acts from the other is common in day-to-day life [73] hindering detection of SV. Heteronormativity also influences social perceptions of who can be considered as a possible perpetrator, causing overlooking of female aggressors [46, 47, 56].

Consequences of CRSV are exacerbated by harmful gender norms, together with cultural and social expectations and attitudes that support violence [74, 75], creating an environment that tolerates and justifies GBV. Gender must be understood as a power dynamic which should be integrated in the analysis of conflict, also in the dimension of violence normalization [8], which is highlighted by the parallelism occurring between heteronormativity and conflict dynamics [76]. Moreover, harmful gender norms, as well as cultural and social attitudes that support violent acts, are associated with perpetration of violent behaviors, including SV [75]. The findings of the present review confirm that CRSV takes place in an enabling environment, characterized by widespread use of sexual and general violence. Its extent opens us the possibility to make a correlation with the phenomenon of overkilling, that is, when the number or the severity of the wounds inflicted on the victim’s body goes beyond that of fatal injuries [40, 77]. Here, the level of violence employed was beyond the one necessary to cause harm from a causal perspective with regards to contextual (e.g., war, instability, gender inequality) and human factors (perpetrators, family and community members, and providers), to the time span when it occurred (before, during, and in the aftermath of CRSV), and type of offenses sustained by victims (e.g., SV, other personal and property offenses, including physical violence), creating an interlinking chain of violence. Exploitative, controlling and violence-related actions [78] took place, and in some cases, physical violence occurring during sexual assault involved the use of force (e.g., employing blunt, natural, and sharp edge weapons), as well as mutilations and killings, resonating with patterns of sexual and sexualized homicides [78] and femicides [79]. CRSV constitutes an objectification and commodification of the victim, potentially up to the point of disposal [79] and is only one of the criminal activities perpetrated by an offender, with a possible “functional” use as a method of punishment [80], or performed as a form of entertainment, and symbolizing “dominance and power” over victims [80]. For these reasons, we propose to use the term “ultraviolence” to refer to the overall offenses suffered by people subjected to CRSV. This encompasses the conflict-related non-sexual violence, as well as interpersonal acts of CRSV and non-sexual violence inflicted during sexual assault, together with sexual and non-sexual forms of violence and discrimination perpetrated at a social and institutional level, including in healthcare, causally related to CSRV and affecting survivors in its aftermath.

Stigmatization is a social phenomenon and a form of violence that causes the marginalization of specific groups or individuals who are seen as deviating from social norms [4], and is characterized by labeling, stereotyping, separation, status loss, and discrimination of a person enabled by power dynamics [81]. In the included studies, stigmatization at individual level appeared through internalized stigma [82], highlighted by the psychological consequences of CRSV [41, 42, 45, 47, 48, 52, 54, 55, 58, 61, 62], as well as avoidance stigma [82], characterized by avoiding seeking care for fear of negative attitudes from health workers [41, 48, 52, 58, 60–62], which can worsen adverse consequences of SV. External stigma [82] was enacted by family and community members on survivors [41–43, 45, 46, 48, 50, 51, 55, 57, 58, 60, 61, 63], emerging through the social negative outcomes, and by providers [42, 49, 61, 62], acting as a barrier to access to care, and negatively influenced health outcomes [83], in one case leading to permanent disability [44]. Moreover, stigma emerged also on an institutional level [42, 45, 46, 48, 52, 61, 62, 83].

Stigmatization was also intersectional in terms of co-existence of different individual identities and statuses (e.g., real or perceived STIs positivity, having a termination or deciding to continue the pregnancy, sexual orientation, engagement in sex work, being a member of a specific ethnic group, disability) [41, 42, 44, 50, 51, 55, 57, 58, 61, 62, 84].

Perpetrators are aware of the stigmatizing nature of CRSV and use it as a weapon to cause long-term consequences, including weakening of communities, who later on ostracize survivors in many ways [4], including physical and social rejection, or denial of resources, creating a continuum of violence, as highlighted in the included studies. Stigmatization, combined with gender and sexual norms justifying it, as well as a violence-enabling environment provoked by conflict, encouraged some survivors to detach from their community or forcibly migrate [11, 42, 44–46, 48, 49, 52, 53, 58–61]. The continuum of violence, including sexual assault, protracts after the first occurrence of CRSV, and can extend to settings differing from the home country of migrants (e.g., at crossing of borders, in transit, in refugee settlements, and in the host country) [11]. Migrant survivors were subjected to institutional violence, in the form of lack of trauma-informed and gender-sensitive asylum application interviews [53]. Challenges affecting survivors of CRSV intersected with those connected to migration and were exacerbated by barriers to access to care connected to the legal status, language barriers, and financial difficulties [42, 45, 48, 52, 53].

In the context of access to care, stigma acted as a barrier from multiple perspectives. At times, the secrecy attributed by cisgender female survivors to victimization [58, 60, 61], together with fear of negative reactions from healthcare workers [41, 48, 52, 58, 60, 61] deterred care-seeking. However, this behavior was encouraged by socially accepted gender norms, internalized by survivors. Acceptance of GBV or lack of awareness of the extent of the issue, was expressed at institutional level by lack of GBV-specific training and services [45, 48]; moreover, in some contexts there was lack of gender- and trauma-sensitive protocols, with official reporting being forced upon survivors to access care [46, 61]. Stigmatization and victim-blaming from providers were also reported [48, 49, 62]. Discrimination experienced by cisgender male survivors in healthcare facilities based its foundation on institutionalized stigma, for example lack of awareness and training of staff, as well as lack of services and protocols targeting male survivors [42, 52, 62], and narrow definitions of SV (e.g., restricted to penetrative acts) [42]. These resulted in failure to recognize and document CRSV affecting men [42], and in categorization of assault as a consensual sexual interaction, criminalizing the victim [62]. Moreover, survivors were stigmatized by providers, not believed [42], or victim blamed [62], due to homophobic, racist, and xenophobic attitudes, ableism, and sex worker stigma because of real or perceived identities [42, 62]. Worryingly, in some contexts, these attitudes escalated into support of rape myths [62], forced and non-consensual reporting [62], and revictimization, meaning SV perpetrated from health professionals [62], and verbal and sexual abuse against gay survivors [62].

The findings of the present review reflect the gap of existing peer reviewed literature on CRSV victimization of transgender and gender diverse survivors. Although being at increased risk of CRSV [1], the prevalence of this kind of violence on these populations is “largely unknown” [85], and their condition is usually analyzed together with that of cisgender men and boys subjected to CRSV [1], with data about LGBTIQ + survivors presented in aggregated form [1].

### Strengths and limitations

This review has some key strengths. An in-depth and systematic analysis was conducted, presenting the findings according to the BPS framework, and efforts were made to capture the different outcomes of various kinds of CRSV in a gender-inclusive lens, employing a broad definition of conflict and of CRSV. Moreover, having analyzed only qualitative evidence, although less common in reviews, gave the possibility to call attention to elements which could have been overlooked in quantitative analysis, and contributed to highlighting the significance survivors’ testimonies carry in understanding challenges faced in the recovery process, including barriers to access to care.

This review has some limitations. First, it relies on qualitative data, and for this reason different results which might have emerged through quantitative analysis were excluded. For the same reason, it only captures issues that participants to the included studies wanted to disclose to researchers collecting data, which might be limited due to the stigmatizing nature of CRSV. Not all the included studies have reported results in a gender-inclusive form, especially for what concerns the experiences of transgender and gender diverse survivors, possibly affecting the quality of the reported findings in this review. Moreover, due to the search strategy employed, gray literature was excluded. No quality appraisal was performed.

### Recommendations

From a research perspective, we recommend that data should be inclusively gender disaggregated [86], also in qualitative literature. We equally encourage researchers in this field to avoid definitions of SV that rely on heteronormative standards, and risk suggesting a hierarchical categorization. Future research focused on other themes, such as the management of victims of CRSV, should apply a survivor-centered approach to the issue.

From a practical perspective, the findings of this review suggest that healthcare professionals should receive gender-inclusive training on detection, treatment, and referral for GBV cases [66], including CRSV. Moreover, judging the validity of survivors’ accounts goes beyond healthcare workers’ duty. Rather, in clinical examinations, we encourage providers to treat survivors as patients, trusting their history of offense, and understanding which interventions are most appropriate for them [3]. On the same occasion, collection and preservation of medico-legal evidence and of forensic specimens should be conducted by trained personnel [3, 87]. Patients should be provided with a copy of documentary evidence of the crime they were subjected to, as this may be especially useful for specific populations, such as asylum seekers, whose ability to access to different forms of humanitarian assistance, or to apply for asylum and non-refoulement can be improved by provision of medical evidence of torture, cruel, or inhuman treatment or punishment [87]. Lack of evidence should be considered, given that access to care might occur days, weeks, or months after the event, and should not imply fabrication [87]. In any case, clinical and forensic examination should be conducted only after having explained the procedure and reporting laws to the patient and having consequently obtained informed consent [1, 3, 87].

On an institutional level, gender-specific services and protocols for the management of survivors of CRSV should be implemented [66], in order to meet the needs of all those who were subjected to it, following a “survivor-centered approach” [25], with the awareness that GBV is a SRH issue that affects the overall well-being of victims. Moreover, comprehensive and gender-inclusive sexuality education should be enabled [88], targeting individuals, communities [66], and providers [89], and encompassing neglected areas such as gender equality and GBV [66], in order to promote social change and avoid stigmatization of SRH and of CRSV [66], in line with the United Nations’ 2030 Sustainable Development Goals, in particular number three—Healthy Lives and Well-Being, number five—Gender Equality, and number 16—Peaceful and Inclusive Societies [90]. In addition, we encourage the implementation of gender-specific guidelines and protocols focusing on cisgender male, transgender, and gender diverse populations [66]. Moreover, although recognizing that in some States mandatory reporting was introduced to protect victims from further abuse [87], we call for awareness of the potential detrimental effect of non-consensual mandatory reporting from physicians, in settings where this practice might lead to criminalization of victims and hinder their access to care [87, 91]. Governments in conflict-affected States, as well as in transit and host countries, should be held accountable for the protection and fulfillment of SRHRs of all survivors of CRSV (cisgender female and male, transgender and gender diverse), as well as for the translation of policies targeting health systems and other sectors (e.g., legal and justice) into institutionalized programs and services targeting victims’ needs, and for adequately funding these initiatives [66].

## Conclusion

This systematic review described the adverse consequences of CRSV on the physical, psychological, and social dimensions of survivors, following the BPS model. The negative outcomes mentioned in more studies were pregnancy, manifestations of groups of symptoms attributable to PTSD, and stigma. This review contributed to analyzing the condition of survivors of CRSV, exacerbated by barriers to access to care, and giving space to individual experiences. Qualitative evidence proved to be a crucial component for understanding the gendered negative effects of CRSV, and for recognizing it as a sexual and reproductive health issue. Sexuality education targeting individuals, communities, and providers could help challenging gender norms and roles, as well as GBV. Governments should translate health policies into concrete action targeting survivors of CRSV.

### Supplementary Information

**Additional file 1.** Search strings per each database. Description of data: Search strings used to retrieve articles from PubMed, Scopus and PsychArticles.

**Additional file 2.** Extraction sheet. Extraction sheet used to collect data from retrieved articles.

**Additional file 3: Table 2.** Characteristics of the included studies. Table reporting characteristics of the included studies (study type and methodology, information about the population).

**Additional file 4: Table 3.** Summary of information about CRSV experienced by survivors. Table reporting information about CRSV experienced by survivors in the included studies.

## Data Availability

The datasets used and/or analyzed during the current study are available from the corresponding author on reasonable request.

## Abbreviations

B: Biological
BPS: Biopsychosocial
CF: Cisgender female
CM: Cisgender male
CRSV: Conflict-related Sexual Violence
DRC: Democratic Republic of Congo
FGD: Focus Group Discussion
GBV: Gender-based violence
HIV: Human Immunodeficiency Virus
IPV: Intimate Partner Violence
NGO: Non-Governmental Organization
P: Psychological
PCATI: Public Committee Against Torture in Israel
PRISMA: Preferred Reporting Items for Systematic reviews and Meta-Analyses
PTSD: Post-Traumatic Stress Disorder
S: Social
SRH: Sexual and Reproductive Health
SRHR: Sexual and Reproductive Health and Rights
SRR: Sexual and Reproductive Rights
STIs: Sexually transmitted infections
SV: Sexual Violence
SVRP: Sexual violence-related pregnancy
TGD: Transgender and Gender Diverse
