# Supplementary material for: Negative consequences of conflict-related sexual violence on survivors: a systematic review of qualitative evidence
Source: Int J Equity Health. 2023 Oct 27;22:227. doi: 10.1186/s12939-023-02038-7 (PMC10612192; doi:10.1186/s12939-023-02038-7)
Supplement: Supplementary file 1 — Additional file 1. Search strings per each database. Description of data: Search strings used to retrieve articles from PubMed, Scopus and PsychArticles. [file 12939_2023_2038_MOESM1_ESM.docx]

**Additional file 1.** Search strings.

| **Database** | **Retrieved records** | **Search string** |
| --- | --- | --- |
| PubMed | 888 | ("sex offenses"[MeSH Terms] OR "sexual violence*"[Title/Abstract] OR "sexual assault*"[Title/Abstract] OR "sexual abuse*"[Title/Abstract]) AND ("refugee*"[Title/Abstract] OR "asylum"[Title/Abstract] OR "fragile setting*"[Title/Abstract] OR "fragile state*"[Title/Abstract] OR "fragile context*"[Title/Abstract] OR "war"[Title/Abstract] OR "conflict*"[Title/Abstract] OR "fighting*"[Title/Abstract] OR "terrorism"[Title/Abstract]) |
| Scopus | 2922 | (TITLE-ABS-KEY ( "sex offenses"  OR  "sexual violence*"  OR  "sexual assault*"  OR  "sexual abuse*")  AND  TITLE-ABS-KEY ( "refugee*"  OR  "asylum"  OR  "fragile setting*"  OR  "fragile state*"  OR  "fragile context*"  OR  "war"  OR  "conflict*"  OR  "fighting*"  OR  "terrorism" ) )  AND  PUBYEAR  >  2012 |
| PsycArticles (PsychNet) | 144 | **Any Field**: (“sexual violence*” OR “sexual abuse*” OR “sexual assault*”) *AND* **Any Field**: (“war*” OR “conflict*” OR “fighting*” OR “terrorism” OR “refugee*” OR “asylum” OR “fragile setting*” OR “fragile state*” OR “fragile context*”) *AND* **Year**: 2013 *To* 2023 |
