# Supplementary material for: Negative consequences of conflict-related sexual violence on survivors: a systematic review of qualitative evidence
Source: Int J Equity Health. 2023 Oct 27;22:227. doi: 10.1186/s12939-023-02038-7 (PMC10612192; doi:10.1186/s12939-023-02038-7)
Supplement: Supplementary file 2 — Additional file 2. Extraction sheet. Extraction sheet used to collect data from retrieved articles. [file 12939_2023_2038_MOESM2_ESM.docx]

**Additional file 2.** Extraction sheet.

| **General information of the article** | Title |
| --- | --- |
|  | Authors |
|  | Publication year |
|  | Language |
|  | Country where the study was conducted |
| **Study design** | Study period |
|  | Objective of the study |
|  | Study type |
|  | Methodology |
|  | Population |
| **Information about the survivor’s experience** | Gender |
|  | Sexual orientation |
|  | Type of migrant |
|  | Home country |
|  | Host country |
| **CRSV** | Geographical area where the conflict occurred |
|  | Kind of conflict |
|  | Kind of CRSV |
|  | Other types of co-occurring GBV and other types of violence |
|  | Location where CRSV occurred |
|  | Perpetrators |
|  | Physical consequences |
|  | Psychological consequences |
|  | Social consequences |
|  | Kind of services accessed/the survivor expressed willingness to access |
|  | Barriers to access to care |
| **Limitations** | Study limitations (authors of the studies) |
|  | Study limitations (authors of the review) |
| **Quotes** |  |
