# Supplementary material for: Negative consequences of conflict-related sexual violence on survivors: a systematic review of qualitative evidence
Source: Int J Equity Health. 2023 Oct 27;22:227. doi: 10.1186/s12939-023-02038-7 (PMC10612192; doi:10.1186/s12939-023-02038-7)
Supplement: Supplementary file 3 — Additional file 3: Table 2. Characteristics of the included studies. Table reporting characteristics of the included studies (study type and methodology, information about the population). [file 12939_2023_2038_MOESM3_ESM.docx]

**Table 2.** Characteristics of the included studies.

| **First author, year** | **Study type and methodology** | **Population** | | | | | | |
| --- | --- | --- | --- | --- | --- | --- | --- | --- |
|  |  | **Service Providers** | **Survivors** | **Characteristics of survivors** | | | | |
|  |  |  |  | **Cisgender female** | **Cisgender male** | **Transgender and gender diverse** | **Migrants** | **Migration status and country of origin** |
| Scott, (2018). | Qualitative. Semi-structured interviews. |  | X | X |  |  |  |  |
|  |  |  |  |  |  |  |  |  |
| Onyango, (2016). | Qualitative. Semi-structured interviews. |  | X | X |  |  |  |  |
| Kansiime, (2017). | Qualitative. Semi-structured interviews. | X | X |  | X |  | X | Refugees, persons of concern.  Congo. |
| Palattiyil and Sidhva, (2015). | Qualitative. In-depth interviews. |  | X | X | X |  | X | Asylum seekers.  Burundi, Ethiopia, Ivory Coast, Kenya, Malawi, Nigeria, South Africa, Tanzania, Zimbabwe. |
| Yagi, (2022). | Qualitative. Semi-structured interviews. |  | X |  | X |  |  |  |
| Kohli, (2013). | Qualitative. In-depth interviews, focus group discussions (FDGs). |  | X | X |  |  |  |  |
| Tenaw, (2022). | Mixed methods. In-depth interviews. |  | X | X |  |  |  |  |
| Green, (2022). | Qualitative. Semi-structured interviews. | X |  | X | X | T, GD | X | Refugees.  Myanmar. |
| Al Issa and Beck, (2020). | Qualitative. Interviews. |  | X | X |  |  |  |  |
| Burkhardt, (2016). | Qualitative. Semi-structured interviews. |  | X | X |  |  |  |  |
| Byrskog, (2014). | Qualitative. Semi-structured interviews. |  | X | X |  |  | X | Refugees.  Somalia. |
| Keygnaert, (2014). | Qualitative. In-depth interviews. |  | X | X | X |  | X | Undocumented migrants.  Cameroun, Congo, DRC, Ivory Coast, Mali, other unspecified Sub Saharan countries. |
| Sager, (2016). | Qualitative. Interviews. |  | X | X |  |  | X | Asylum seeker.  Kosovo. |
| Weishut, (2015). | Qualitative. Retrospective analysis of archive files. |  | X |  | X |  |  |  |
| Woldetsadik, (2022). | Qualitative. In-depth interviews, FDGs. |  | X | X |  |  |  |  |
| Chynoweth, (2020). | Qualitative. Semi-structured interviews, FDGs, document review. |  | X |  | X | T | X | Refugees, migrants.  DRC, Myanmar, Somalia, South Sudan. |
| Dossa, (2014). | Qualitative. Semi-structured interviews. |  | X | X |  |  |  |  |
| Haar, (2019). | Qualitative. Semi-structured interviews, physical examination. |  | X | X | X |  | X | Refugees.  Myanmar. |
| Wirtz, (2014). | Qualitative. In-depth interviews, FDGs. | X | X | X |  |  | X | Internally displaced persons.  Colombia. |
| Corboz, (2022). | Qualitative. Semi-structured interviews. | X | X |  | X |  |  |  |
| Atim, (2018). | Mixed methods. In-depth interviews. |  | X | X |  |  |  |  |
| Krause, (2015). | Qualitative. Semi-structured interviews, FDGs, participatory observation, structured, and semistructured expert interviews, ero-epic dialogues or unstructured FGDs. | X | X | X | X |  |  | Refugees.  DRC. |
| Wirtz, (2013). | Qualitative. Semi-structured interviews, FDGs. | X |  | X |  |  |  | Refugees.  Burundi, DRC, Eritrea, Somalia, Sudan. |
