# Supplementary material for: Negative consequences of conflict-related sexual violence on survivors: a systematic review of qualitative evidence
Source: Int J Equity Health. 2023 Oct 27;22:227. doi: 10.1186/s12939-023-02038-7 (PMC10612192; doi:10.1186/s12939-023-02038-7)
Supplement: Supplementary file 4 — Additional file 4: Table 3. Summary of information about CRSV experienced by survivors. Table reporting information about CRSV experienced by survivors in the included studies. [file 12939_2023_2038_MOESM4_ESM.docx]

**Table 3.** Summary of information about CRSV experienced by survivors.

| **Kind of CRSV** | **CF** | **CM** | **TGD** |
| --- | --- | --- | --- |
| **Direct CRSV** | | | |
| **Rape** | | | |
| Penile penetration (vaginal) | X |  |  |
| Penile penetration (anal) |  | X |  |
| Penetration with fingers (vaginal) | X |  |  |
| Penetration with fingers (anal) | X |  |  |
| Penetration with blunt objects |  | X |  |
| Gang rape | X |  |  |
| Multiple perpetrator rape | X |  |  |
| **SV specific to pregnant women** | | | |
| Forced pregnancy | X |  |  |
| Forced abortion | X |  |  |
| **Mutilating injuries** | | | |
| Breast mutilation | X |  |  |
| Castration |  | X |  |
| **Genito-anal injuries** | | | |
| Genital sharp force injuries | X |  |  |
| Torture directed at genito-anal area (with use of blunt, or electrical force, or irritant agents) |  | X |  |
| **Injuries on other erogenous sites** | | | |
| Blunt force injury to breasts | X |  |  |
| **Attempted SV** | | | |
| Attempted rape | X |  |  |
| Attempted gang rape | X |  |  |
| **Exploitation for sexual purposes** | | | |
| Sexual slavery | X |  |  |
| Forced prostitution | X |  |  |
| Trafficking | X |  |  |
| Forced transactional sex for having access to food, shelter, security, pass-through, transportation. | X |  |  |
| **Forced marriage** | | | |
| Forced marriage with stranger decided by armed forces | X |  |  |
| Forced marriage with perpetrator of CRSV | X |  |  |
| **Forced nudity** | | | |
| Forced nudity | X | X |  |
| **Unwanted sexual touching** | | | |
| Unwanted sexual touching (breasts, intimate parts) | X |  |  |
| Inappropriate body searching | X |  |  |
| **Humiliating acts** | | | |
| Perpetrator urinated on survivor’s face and body |  | X |  |
| **SV not involving** **physical contact** | | | |
| **Verbal SV** | | | |
| Comments | X |  | X |
| Homophobic comments |  | X |  |
| Humiliation |  | X |  |
| Humiliation referred to members of the family |  | X |  |
| **Non verbal SV** | | | |
| Gestures | X |  |  |
| Simulated rape |  | X |  |
| **Threats** | | | |
| Threats of rape | X |  |  |
| Threats of rape of a family member |  |  | X |
| Threat of sexual violence | X | X |  |
| Threat of sexual violence of a family member |  | X |  |
| **Reiteration of SV** | | | |
| Revictimization by perpetrator after reporting | X |  |  |
| **Witnessed CRSV** | | | |
| **Forced witnessing** | | | |
| Forced witnessing of SV |  | X |  |
| Forced witnessing of SV on family members | X |  |  |
| Forced witnessing of rape |  | X |  |
| Forced witnessing of rape of family members or acquaintances | X |  |  |
| Forced witnessing of rape of other in transit, unknown to survivor | X |  |  |
| **Witnessing** | | | |
| Witnessing of SV on adults | X |  |  |
| Witnessing of SV on minors | X |  |  |
| Witnessing of rape | X |  |  |
| Witnessing of others being raped and murdered | X |  |  |
| Witnessing of minors being raped | X |  |  |
| Witnessing of forced nudity |  | X |  |
